# Supplementary material for: Effects of high intensity interval training versus moderate intensity continuous training on exercise capacity and quality of life in patients with heart failure: A systematic review and meta-analysis
Source: PLoS One. 2023 Aug 17;18(8):e0290362. doi: 10.1371/journal.pone.0290362 (PMC10434865; doi:10.1371/journal.pone.0290362)
Supplement: S1 File — (DOCX) [file pone.0290362.s002.docx]

### Supplementary Material 1. Search strategy

*Pubmed*

#1 "High-Intensity Interval Training"[MeSH Terms] -------------------------------------------------------1,947

#2 "High Intensity"[Title/Abstract] AND ("intermittent exercise*"[Title/Abstract] OR "interval training*"[Title/Abstract]) -------------------------------------------------------------------------------------3,415

#3 "Sprint Interval"[Title/Abstract] AND "training*"[Title/Abstract] -------------------------------------461

#4 ("interval*"[Title/Abstract] OR "intermittent*"[Title/Abstract]) AND ("training*"[Title/Abstract] OR "exercise*"[Title/Abstract]) ----------------------------------------------------------------------------------47,277

#5 #1 OR #2 OR #3 OR #4 ------------------------------------------------------------------------------------47,476

#6 "continuous training*"[Title/Abstract] OR "continuous exercise*"[Title/Abstract] OR "continuous aerobic exercise*"[Title/Abstract] OR "continuous aerobic training*"[Title/Abstract] -----------------2457

#7 "Heart Failure"[MeSH Terms] --------------------------------------------------------------------------141,957

#8 ("heart*"[Title/Abstract] OR "cardiac*"[Title/Abstract] OR "myocard*"[Title/Abstract]) AND ("fail*"[Title/Abstract] OR "insuff*"[Title/Abstract] OR "incompet*"[Title/Abstract] OR "decomp*"[Title/Abstract]) ---------------------------------------------------------------------------------314,994

#9 "heart"[Title/Abstract] AND "attack"[Title/Abstract] --------------------------------------------------8,944

#10 #7 OR #8 OR #9 -----------------------------------------------------------------------------------------350,730

#11 #5 AND #6 AND #10 ----------------------------------------------------------------------------------------107

Date Run: 12/11/2022

*Cochrane Central Register of Controlled Trials*

#1 MeSH descriptor: [High-Intensity Interval Training] explode all trees ----------------------------------700

#2 ("High Intensity"):ti,ab,kw AND (("Interval Training*"):ti,ab,kw OR ("Intermittent Exercise*"):ti,ab,kw) -------------------------------------------------------------------------------------------2,959

#3 ("Sprint Interval"):ti,ab,kw AND (Training*):ti,ab,kw ---------------------------------------------------238

#4 ((interval*):ti,ab,kw OR (intermittent*):ti,ab,kw) AND ((training*):ti,ab,kw OR (exercise*):ti,ab,kw)-------------------------------------------------------------------------------------------19,554

#5 #1 OR #2 OR #3 OR #4 ------------------------------------------------------------------------------------19,554

#6 ("continuous training*"):ti,ab,kw OR ("continuous exercise*"):ti,ab,kw OR ("continuous aerobic exercise*"):ti,ab,kw OR ("continuous aerobic training*"):ti,ab,kw---------------------------------------1,404

#7 MeSH descriptor: [Heart Failure] explode all trees -----------------------------------------------10,621

#8 ((heart*):ti,ab,kw OR (cardiac*):ti,ab,kw OR (myocard*):ti,ab,kw) AND ((fail*):ti,ab,kw OR (insuff*):ti,ab,kw OR (incompet*):ti,ab,kw OR (decomp*):ti,ab,kw) ----------------------------------50,907

#9 (heart):ti,ab,kw AND (attack):ti,ab,kw --------------------------------------------------------------------3,035

#10 #7 OR #8 OR #9 -------------------------------------------------------------------------------------------52,921

#11 #5 AND #6 AND #10 ----------------------------------------------------------------------------------------156

Date Run: 12/11/2022

*Embase*

#1 'high intensity interval training'/exp -----------------------------------------------------------------------3,955

#2 'high intensity':ab,ti AND ('interval training*':ab,ti OR 'intermittent exercise*':ab,ti) --------------3,894

#3 'sprint interval':ab,ti AND training*:ab,ti -------------------------------------------------------------------419

#4 (interval*:ab,ti OR intermittent*:ab,ti) AND (training*:ab,ti OR exercise*:ab,ti) -----------------59,675

#5 #1 OR #2 OR #3 OR #4 ------------------------------------------------------------------------------------60,402

#6 'continuous training*':ab,ti OR 'continuous exercise*':ab,ti OR 'continuous aerobic training*':ab,ti OR 'continuous aerobic exercise*':ab,ti ---------------------------------------------------------------------------3,343

#7 'heart failure'/exp ------------------------------------------------------------------------------------------619,455

#8 (heart*:ab,ti OR cardiac*:ab,ti OR myocard*:ab,ti) AND (fail*:ab,ti OR insuff*:ab,ti OR incompet*:ab,ti OR decomp*:ab,ti) ------------------------------------------------------------------------498,715

#9 heart:ab,ti AND attack:ab,ti -------------------------------------------------------------------------------14,037

#10 #7 OR #8 OR #9 -----------------------------------------------------------------------------------------809,237

#11 #5 AND #6 AND #10 ----------------------------------------------------------------------------------------195

Date Run: 12/11/2022

*ClinicalTrials.gov*

Condition or disease: “heart failure”
Other terms: randomized
Intervention/treatment: “High-Intensity Interval Training”
Applied filters: interventional, adult, older adult

Results: ----------------------------------------------------------------------------------------------------------------1

ClinicalTrials.gov Identifier: NCT02147613

Date Run: 12/11/2022
