# Supplementary material for: Effects of high intensity interval training versus moderate intensity continuous training on exercise capacity and quality of life in patients with heart failure: A systematic review and meta-analysis
Source: PLoS One. 2023 Aug 17;18(8):e0290362. doi: 10.1371/journal.pone.0290362 (PMC10434865; doi:10.1371/journal.pone.0290362)
Supplement: S2 File — (DOCX) [file pone.0290362.s003.docx]

**Supplementary Material 2. Descriptive statistical analysis of TET for HF and HFrEF with SPSS 21.0**

Normality tests of TET for HF and HFrEF

| **Tests of Normality** | | | | | | | |
| --- | --- | --- | --- | --- | --- | --- | --- |
|  |  | Kolmogorov-Smirnov^a^ | | | Shapiro-Wilk | | |
|  |  | Statistic | df | Sig. | Statistic | df | Sig. |
| HF | HIIT | 0.239 | 16 | 0.015 | 0.906 | 16 | 0.099 |
|  | MICT | 0.149 | 16 | 0.200 | 0.982 | 16 | 0.976 |
| HFrEF | HIIT | 0.251 | 13 | 0.025 | 0.925 | 13 | 0.293 |
|  | MICT | 0.167 | 13 | 0.200 | 0.975 | 13 | 0.945 |

^a^: Lilliefors Significance Correction.

Percentiles distribution of TET for HF and HFrEF

| **Percentiles** | | | | | | | | |
| --- | --- | --- | --- | --- | --- | --- | --- | --- |
|  | | | Percentiles | | | | | |
|  |  |  | 5 | 10 | 25 | 50 | 75 | 90 |
| HF | Weighted Average | HIIT | 453.0 | 513.9 | 1191.0 | 1368.0 | 1440.0 | 2203.2 |
|  |  | MICT | 459.0 | 652.5 | 1134.0 | 1671.0 | 2137.5 | 2552.4 |
| HFrEF | Weighted Average | HIIT | 540.0 | 708.0 | 1194.0 | 1368.0 | 1584.0 | 2246.4 |
|  |  | MICT | 720.0 | 816.0 | 1188.0 | 1650.0 | 2115.0 | 2692.8 |

Histograms of TET for HF and HFrEF


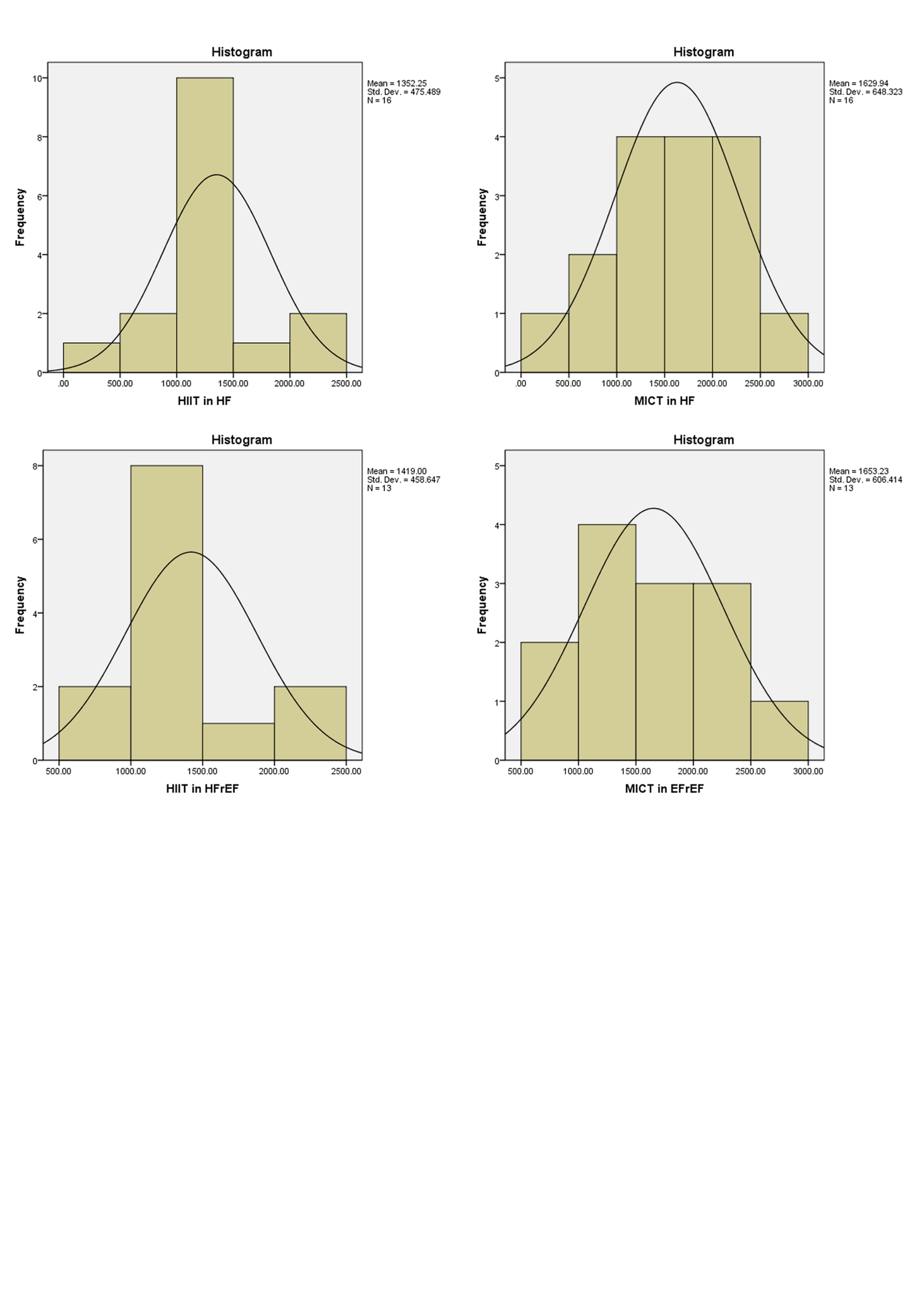


Subgroup of TET for HF and HFrEF

|  | Short time | | Medium time | | | | Long time | |
| --- | --- | --- | --- | --- | --- | --- | --- | --- |
|  | Study | Time (min) | Study | Time (min) | Study | Time (min) | Study | Time (min) |
| Subgroup of TET of HIIT for HF | Angadi et al | 453 | Benda et al | 1200 | Fu et al | 1188 | Iellamo et al (2014) | 1728 |
|  | Besnier et al | 540 | Dimopoulos et al | 1440 | Iellamo et al (2013) | 1407 | Koufaki et al | 2304 |
|  | Papathanasiou et al | 960 | Donelli et al | 1368 | Mueller et al | 1368 | Ulbrich et al | 2160 |
|  |  |  | Ellingsen et al | 1368 | Roditis et al | 1440 |  |  |
|  |  |  | Freyssin et al | 1344 | Wisløff et al | 1368 |  |  |
| Time range (453~2304) | (453~1069) |  | (1070~1686) | | | | (1687~2304) |  |
| Subgroup of TET of MICT for HF | Angadi et al | 495 | Dimopoulos et al | 1440 | Iellamo et al (2014) | 2070 | Freyssin et al | 2880 |
|  | Benda et al | 1080 | Donelli et al | 1692 | Roditis et al | 1440 | Koufaki et al | 2412 |
|  | Besnier et al | 720 | Ellingsen et al | 1692 | Wisløff et al | 1692 | Mueller et al | 2400 |
|  | Papathanasiou et al | 960 | Fu et al | 1296 |  |  | Ulbrich et al | 2160 |
|  |  |  | Iellamo et al (2013) | 1650 |  |  |  |  |
| Time range (495~2880) | (495~1289) |  | (1290~2084) | | | | (2085~2880) |  |
| Subgroup of TET of HIIT for HFrEF | Besnier et al | 540 | Benda et al | 1200 | Fu et al | 1188 | Iellamo et al (2014) | 1728 |
|  | Papathanasiou et al | 960 | Dimopoulos et al | 1440 | Iellamo et al (2013) | 1407 | Koufaki et al | 2304 |
|  |  |  | Ellingsen et al | 1368 | Roditis et al | 1440 | Ulbrich et al | 2160 |
|  |  |  | Freyssin et al | 1344 | Wisløff et al | 1368 |  |  |
| Time range (540~2304) | (540~1127) |  | (1128~1715) | | | | (1716~2304) |  |
| Subgroup of TET of MICT for HFrEF | Benda et al | 1080 | Dimopoulos et al | 1440 | Roditis et al | 1440 | Freyssin et al | 2880 |
|  | Besnier et al | 720 | Ellingsen et al | 1692 | Wisløff et al | 1692 | Koufaki et al | 2412 |
|  | Fu et al | 1296 | Iellamo et al (2013) | 1650 |  |  | Ulbrich et al | 2160 |
|  | Papathanasiou et al | 960 | Iellamo et al (2014) | 2070 |  |  |  |  |
| Time range (720~2880) | (720~1439) |  | (1440~2159) | | | | (2160~2880) |  |
