# Supplementary figures and images for: Effects of high intensity interval training versus moderate intensity continuous training on exercise capacity and quality of life in patients with heart failure: A systematic review and meta-analysis
Source: PLoS One. 2023 Aug 17;18(8):e0290362. doi: 10.1371/journal.pone.0290362 (PMC10434865; doi:10.1371/journal.pone.0290362)

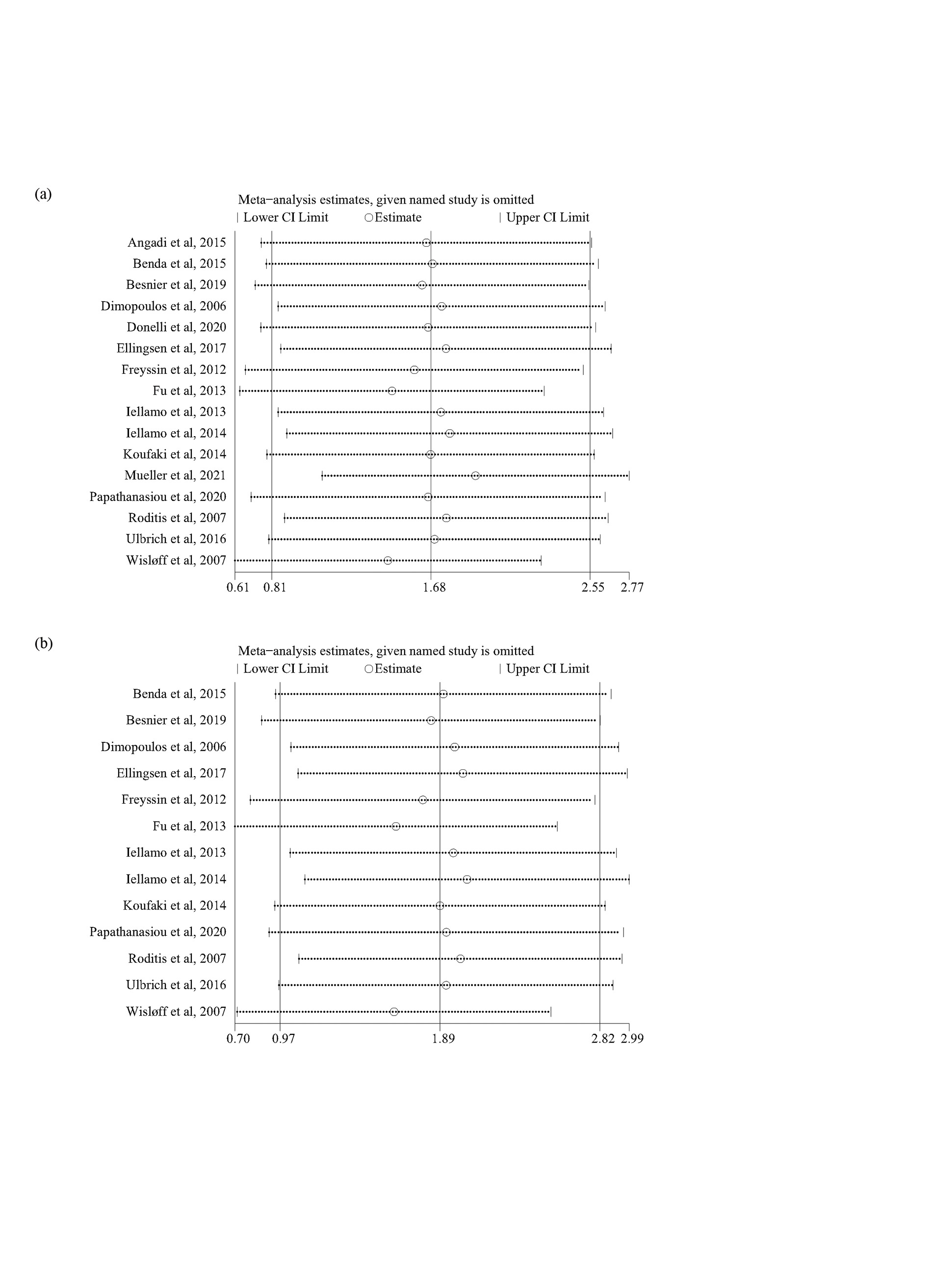

Supplement: S1 Fig — (a) for HF studies; (b) for HFrEF studies. (TIF) [file pone.0290362.s004.tif]

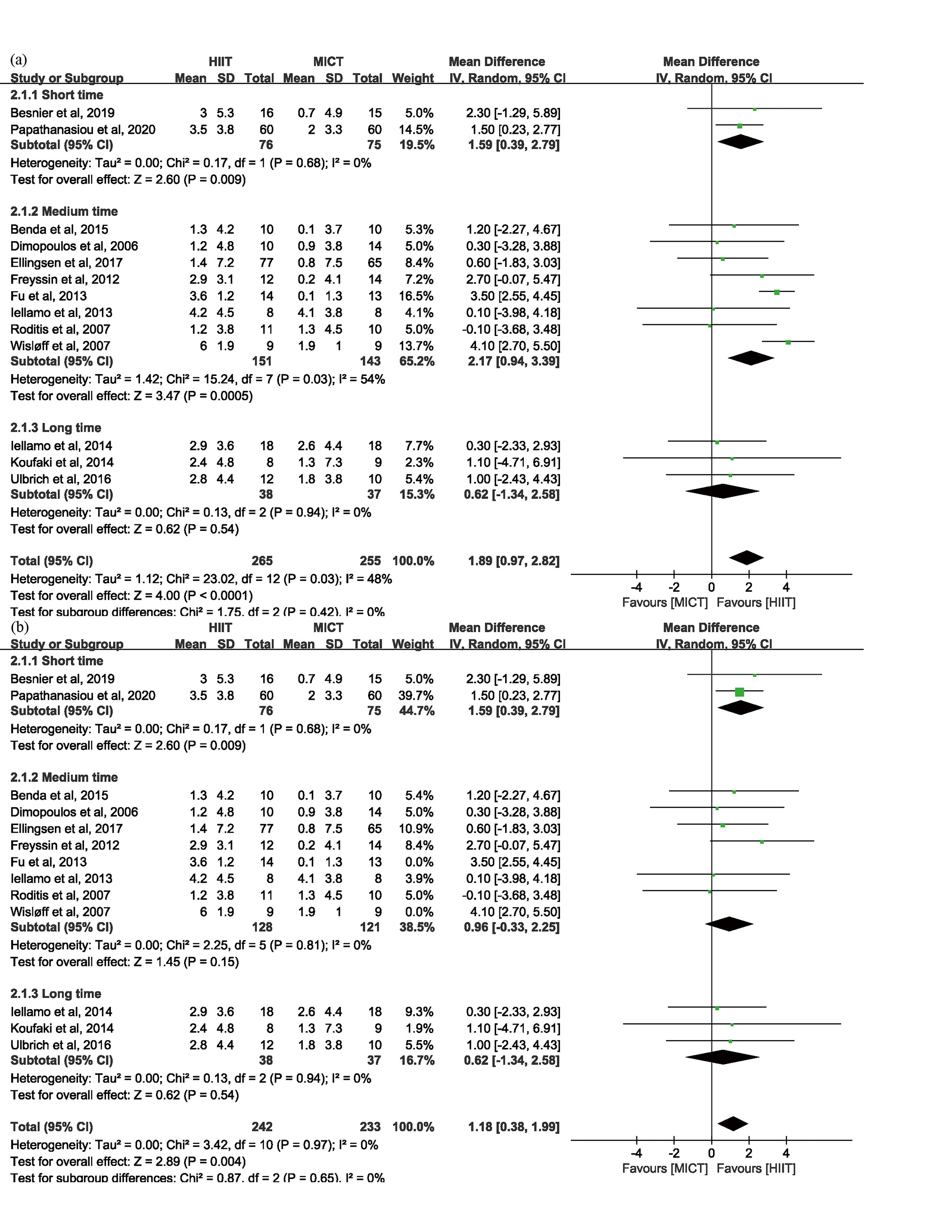

Supplement: S2 Fig — (TIF) [file pone.0290362.s005.tif]

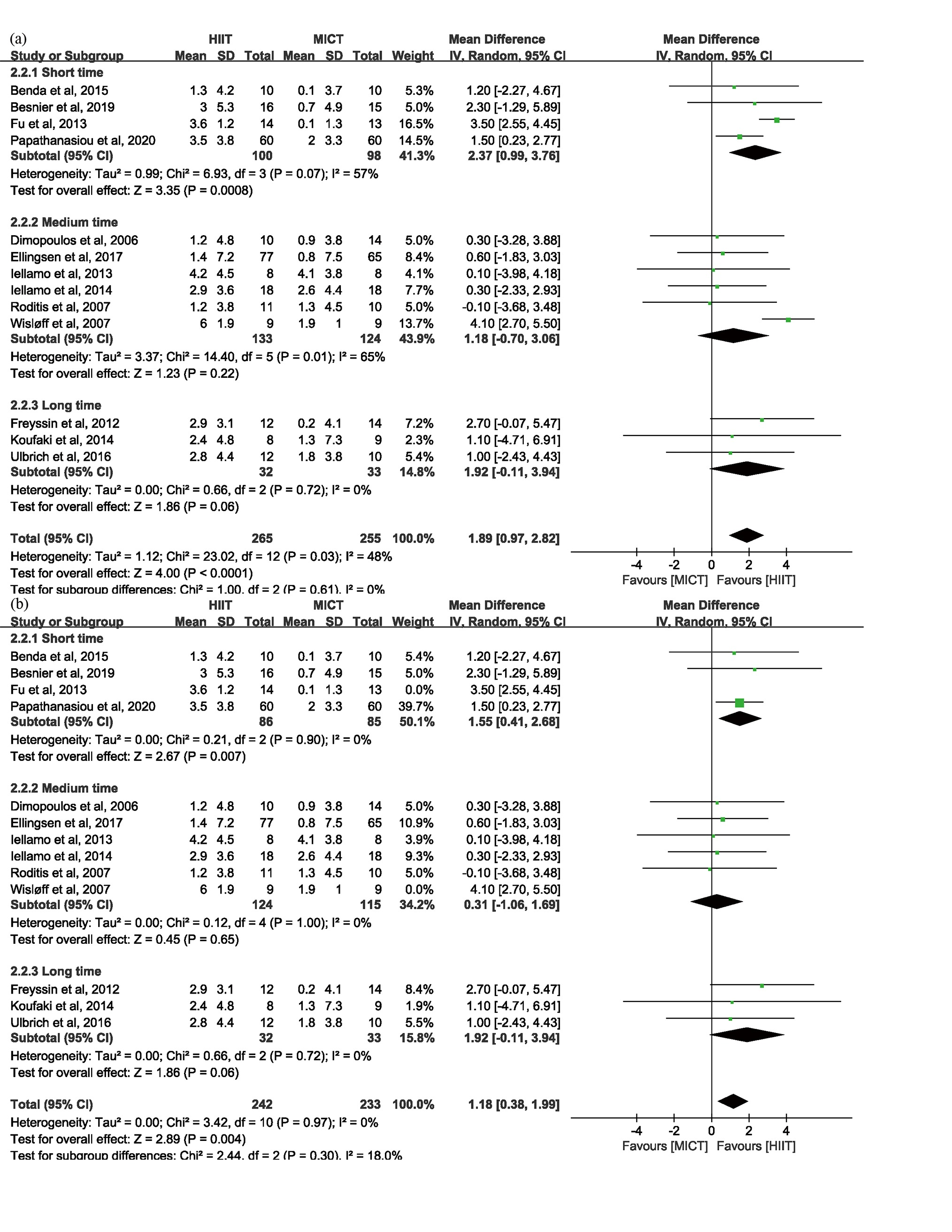

Supplement: S3 Fig — (TIF) [file pone.0290362.s006.tif]

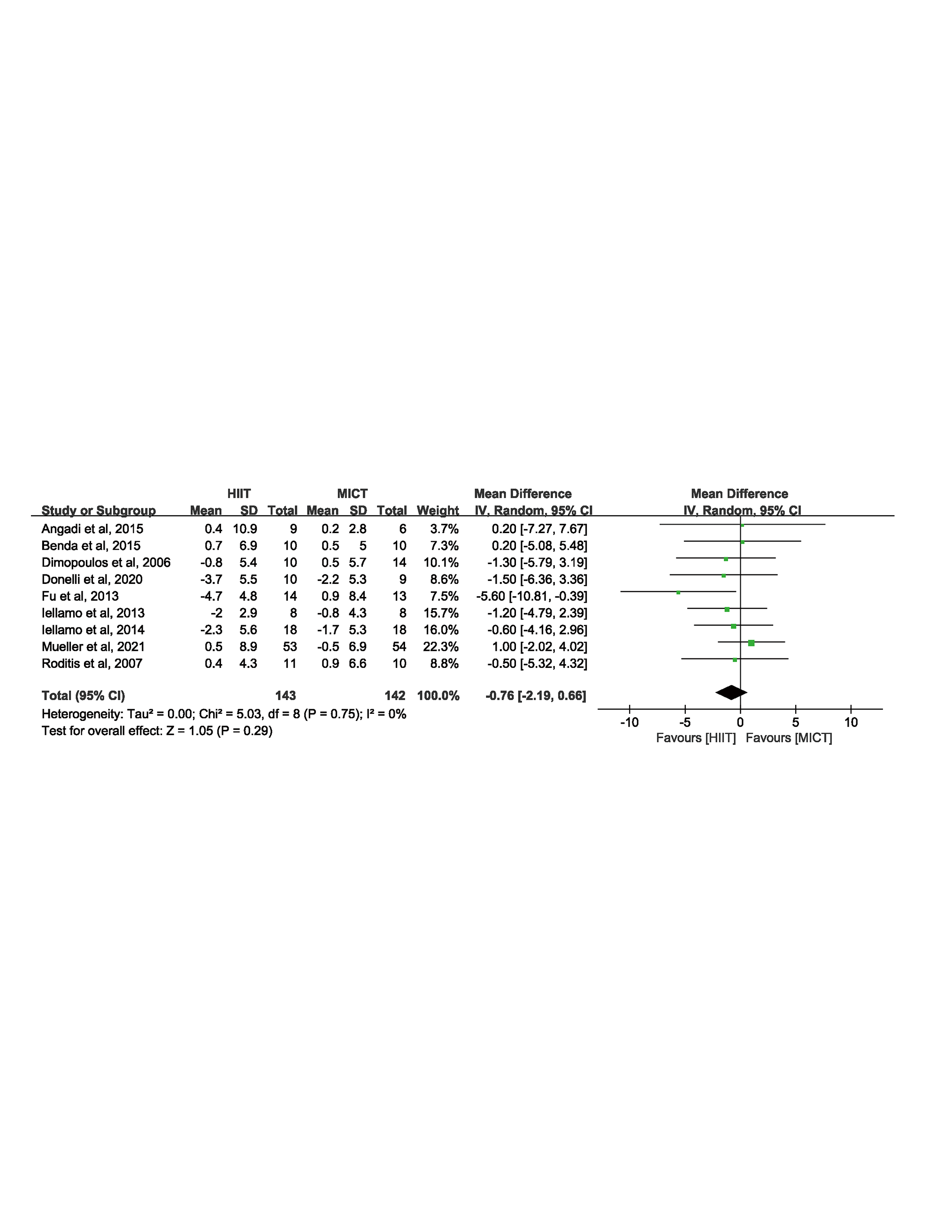

Supplement: S4 Fig — (TIF) [file pone.0290362.s007.tif]

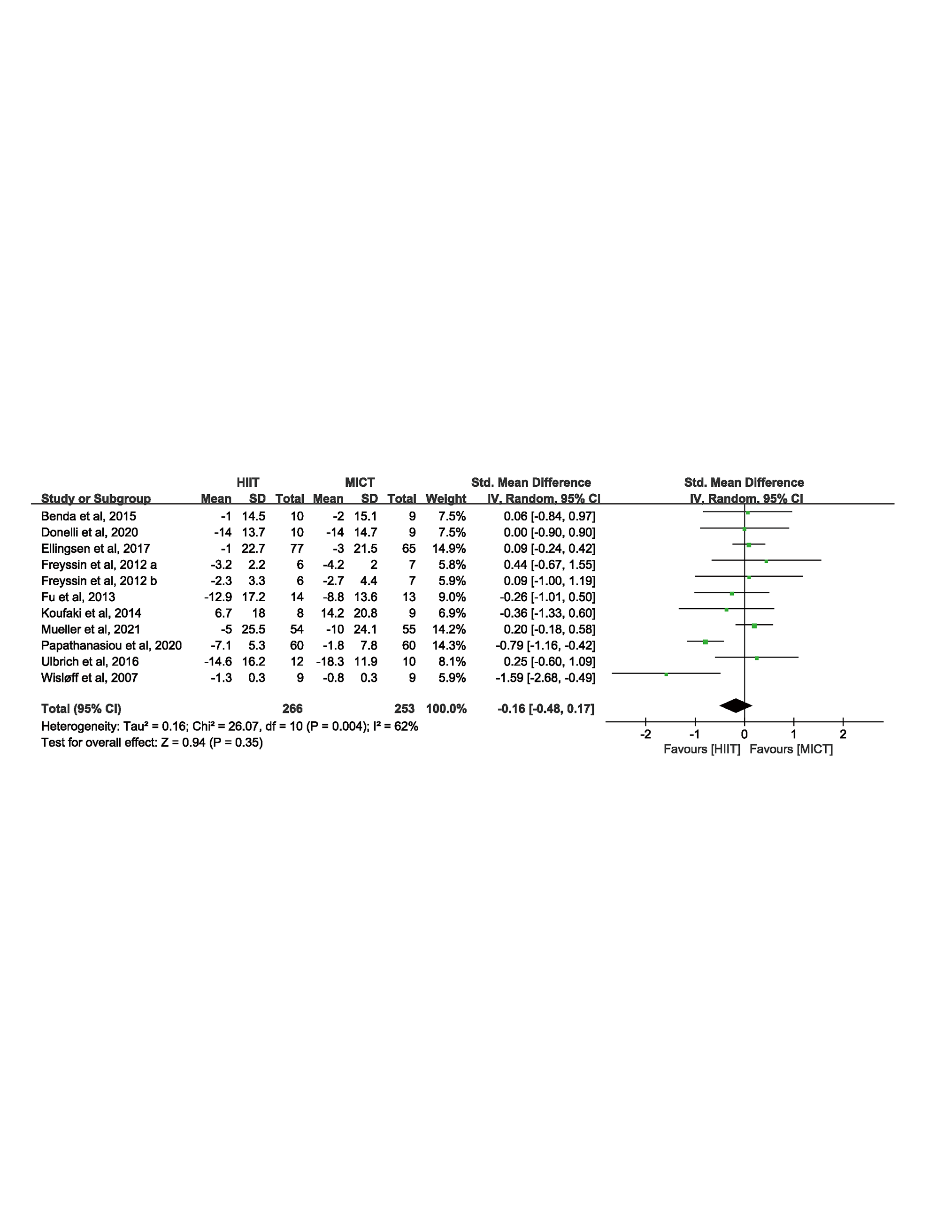

Supplement: S5 Fig — (TIF) [file pone.0290362.s008.tif]
